# Atypical bacterial pneumonia in the HIV-infected population

**DOI:** 10.1186/s41479-017-0036-z

**Published:** 2017-08-25

**Authors:** Breanne M. Head, Adriana Trajtman, Zulma V. Rueda, Lázaro Vélez, Yoav Keynan

**Affiliations:** 1 Department of Medical Microbiology, University of Manitoba, Winnipeg, Canada; 2 Facultad de Medicina, Universidad Pontificia Bolivariana, Medellin, Colombia; 3 Grupo Investigador de Problemas en Enfermedades Infecciosas, Universidad de Antioquia UdeA, Medellin, Colombia; 4 Department of Internal Medicine, University of Manitoba, Winnipeg, Canada; 5 Department of Community Health Sciences, University of Manitoba, Winnipeg, Canada

**Keywords:** Atypical, Bacteria, Pneumonia, HIV, Legionella, Chlamydophila, Mycoplasma, Coxiella, Tropheryma

## Abstract

Human immunodeficiency virus (HIV)-infected individuals are more susceptible to respiratory tract infections by other infectious agents (viruses, bacteria, parasites, and fungi) as their disease progresses to acquired immunodeficiency syndrome. Despite effective antiretroviral therapy, bacterial pneumonia (the most frequently occurring HIV-associated pulmonary illness) remains a common cause of morbidity and mortality in the HIV-infected population. Over the last few decades, studies have looked at the role of atypical bacterial pneumonia (i.e. pneumonia that causes an atypical clinical presentation or responds differently to typical therapeutics) in association with HIV infection. Due to the lack of available diagnostic strategies, the lack of consideration, and the declining immunity of the patient, HIV co-infections with atypical bacteria are currently believed to be underreported. Thus, following an extensive database search, this review aimed to highlight the current knowledge and gaps regarding atypical bacterial pneumonia in HIV. The authors discuss the prevalence of *Chlamydophila pneumoniae*, *Mycoplasma pneumoniae*, *Coxiella burnetii*, *Legionella* species and others in the HIV-infected population as well as their clinical presentation, methods of detection, and treatment. Further studies looking at the role of these microbes in association with HIV are required. Increased knowledge of these atypical bacteria will lead to a more rapid diagnosis of these infections, resulting in an improved quality of life for the HIV-infected population.

## Background

Bacterial pneumonia, the most frequent human immunodeficiency virus (HIV)-associated pulmonary illness, is a common cause of co-morbidity and mortality in the HIV population. Prior to the introduction of combination antiretroviral therapy (cART), bacterial pneumonia infection rates ranged from 3.9–20 cases per 100 person-years in HIV-positive individuals and were predominantly due to opportunistic pathogens such as *Streptococcus pneumoniae, Haemophilus influenzae, Staphylococcus aureus*, as well as acute *Mycobacterium tuberculosis* infections [1–8]. Although bacterial pneumonia rates have decreased since the introduction of cART, rates remain 10 times higher among HIV-infected individuals than in healthy individuals [2, 4, 5, 9, 10]. Additionally, HIV-associated pneumonia remains the most common cause of hospital admission with up to 90 cases per 1000 persons occurring yearly [5, 11–13].

Currently, the diagnosis of pneumonia is based on clinical features and X-ray. The etiological diagnosis, however, is based on empirical data, culture, serology, nucleic acid amplification techniques (NAAT), and bronchoscopy [5, 14]. When choosing any of these diagnostics, a number of points must be considered. Although very informative, empirical data (i.e. patient history, recent travel, intravenous [IV] drug exposure, prior infections, or antibiotic exposure) can only aid in narrowing the scope of infection and is not definitive [5]. In contrast, culture allows bacterial identification and is considered the preferred method in diagnostics. However, incubation periods can be lengthy (depending on the growth rate of the microorganism), not all microbes are cultivable, and the sensitivity of the assay decreases if the patient has had any pre-treatment with antibiotics [15]. Serological tests rely on the patient’s ability to mount an effective antibody response; however, in the case of HIV, this response is greatly reduced. Therefore, depending on the stage of HIV infection, serology may not be clinically useful [16]. Due to the low sensitivities of serology, NAAT (such as polymerase chain reaction [PCR]) is becoming the diagnostic tool of choice for rapid identification of atypical bacteria in respiratory samples of HIV-infected and uninfected individuals [14, 17, 18]. However, although NAAT is highly specific, sensitivity has been shown to vary depending on the patient sample being tested (e.g. nasopharyngeal sample vs. induced sputum) [19, 20]. Moreover, although the presence of NAAT is becoming more prominent in developed nations, it is still not readily available in developing countries. In the event that a definitive diagnosis cannot be reached, more invasive techniques (e.g. bronchoscopy) may be used for sample collection (bronchoalveolar lavage [BAL] or biopsy). Although very beneficial, bronchoscopy is currently underutilized in respect to HIV and advanced immunosuppression, despite it being recommended for patients with low CD4 cell counts (< 200 cells/μl). This underutilization is perhaps due to the fact that patients are too sick to undergo the BAL procedure, or due to the high volume of immunosuppressed patients admitted to hospital [5, 12, 21]. In addition, it can lead to complications such as bleeding and pneumothorax [5, 21].

Despite technological advancements, the etiology of HIV-associated pneumonia is identified less than 60% of the time, thus research looking into atypical pneumonia-causing agents is required [5, 9]. Limited information is available regarding atypical bacterial pneumonia (i.e. pneumonia that does not respond to beta-lactams, one of the antibiotics typically prescribed to pneumonia patients with co-morbidities), with even less information about these infections in HIV [22]. Due to the lack of consideration, the role that atypical bacteria play in disease severity and patient outcome in HIV-associated pneumonia is unknown. Consequently, this review will highlight the bacteria—namely *Chlamydophila pneumoniae*, *Mycoplasma pneumoniae*, *Coxiella burnetii*, *Legionella* species and others—that is responsible for causing atypical bacterial pneumonia in HIV. Specifically, the review explores the current knowledge regarding the prevalence of these microbes in the HIV-infected population, as well as their clinical presentation, methods of detection, and treatment.

### Chlamydophila pneumoniae

*Chlamydophila pneumoniae*, an obligate intracellular pathogen that has caused pulmonary infections all over the world, remains a particular problem in the HIV-infected population [23–26]. Studies by Trinh et al. [27] have demonstrated that *C. pneumoniae* pneumonia rates are as high as 60% in cART-treated HIV-infected children. Likewise, in the adult population, coinfections with HIV have been reported to range from 3 to 39% [28–31]. In general, HIV-associated *C. pneumoniae* pneumonia rates have been shown to be inversely proportional to the patient’s CD4 cell count, occurring at 6.8, 15.7, and 25.2% when CD4 counts are above 500, between 200 and 500, and below 200 cells/μl, respectively [30]. In other words, the rates increase with decreasing CD4, up to a quarter among individuals with advanced HIV and CD4 < 200. In a retrospective analysis [29] of 319 adult pneumonia-infected HIV seropositive individuals, *C. pneumoniae* was cited as a co-pathogen with other microorganisms in approximately 2.2% (*n* = 7) of cases*.*

Much of the research on HIV-associated *C. pneumoniae* pneumonia arises from the post-cART era with little information on the effect of this microorganism in untreated HIV. However, of those that have been reported, it was found that the risk of acquiring *C. pneumoniae* pneumonia was 5 times higher in untreated HIV than in the general population [30, 32, 33]. Regardless of these higher rates, the clinical course of disease is similar among both populations. Disease manifests as an acute respiratory infection (with focal pneumonia, pleural effusion, or bronchitis), although, as the degree of immunosuppression increases, more severe and diffuse interstitial pulmonary involvement and death can occur [29, 32, 34, 35]. Likewise, *C. pneumonia* infection has also been shown to cause chronic infections (e.g. arteriosclerosis or cardiovascular disease) [36].

Diagnosis of HIV-associated *C. pneumoniae* pneumonia is reliant on serology and NAAT. Microimmunofluorescence (MIF), a technique that indirectly measures the *C. pneumoniae-*specific antibody response, requires either single or convalescent serum samples to differentiate between a recent/current infection and a previous one [27, 30, 29, 34]. However, severely immunosuppressed HIV-infected adults (with CD4 counts <200 cells/μL) have been reported to be unable to mount an effective IgG response [25]. Conversely, HIV patients may have an asymptomatic *C. pneumoniae* infection while co-infected with another pneumonia-causing pathogen, which poses another limitation on the utility of this diagnostic test [25, 29]. Consequently, NAAT tests on respiratory specimens (BAL or nasophayngeal swab) are recommended as they have shown promise for *C. pneumonia* diagnosis in HIV [18, 27, 37]. In fact, the United States Food and Drug Administration has approved a BioFire FilmArray NAAT for the detection of both *C. pneumoniae* and *M. pneumoniae* [37].

### Mycoplasma pneumoniae

*Mycoplasma pneumoniae*, the most common *Mycoplasma* respiratory pathogen, accounts for approximately 20% of all pneumonias in the United States (US) general population, and 11.3–21.0% (depending on the method of diagnosis) of all pneumonias in the US HIV-infected population, with higher rates correlated with the degree of immunosuppression [31, 38–40]. Indeed, in a study by Nadagir et al. [41], 18 of the 29 (62%) HIV-positive, *M. pneumoniae*-infected children had a CD4 cell count of <20 cells/μL. Additionally, a depleted CD4 associated with advanced HIV disease has been shown to enhance *M. pneumoniae* establishment in the lungs [40–43]. However, similarly to *C. pneumoniae*, the majority of the data on HIV-associated *M. pneumoniae* lung infections is from treated HIV patients, with minimal information on its effect in untreated HIV.

Clinical manifestations of *M. pneumoniae* pneumonia in HIV are similar to those seen in healthy individuals. Cough (reported in 100% of cases), anemia, arthralgia, dyspnea, and sore throat along with fever, rales, interstitial infiltrates, and lobar pneumonia are most commonly reported, making diagnosis nearly impossible based solely on clinical presentation [40–42, 38].

*M. pneumoniae* diagnosis relies on culture, serology, and NAAT [42, 44]. However, isolation requires up to 3 weeks incubation, limiting the practicality of this method in a clinical setting [40, 41, 38, 45]. Similarly, time is also a limiting factor for serology, as it is dependent on a convalescent serum sample [19]. Moreover, *M. pneumoniae* has been shown to persist within the host, with persistent IgM detectable years after infection [38, 41, 42, 45–50]. Furthermore, due to the fact that up to 20% of healthy individuals do not develop *M. pneumoniae*-specific IgM combined with the impaired immune response associated with HIV infection, immunosuppressed HIV-infected patients may never develop a detectable antibody response altogether, which means this technique is not reliable for diagnosis in this population [38, 51]. In fact, Shankar et al. [40] found that culture was more reliable for diagnosing *M. pneumoniae* infections in HIV-positive individuals since it was able to identify infections in 31% (*n* = 31) of their adult HIV population, while IgM enzyme-linked immunosorbent assay only identified 21% (*n* = 21), highlighting that relying solely on serology could lead to a false negative. Consequently, multiple laboratories have developed NAAT methods (e.g. the BioFire FilmArray NAAT, or real-time PCR) for the detection of *M. pneumoniae*, although the Center for Disease Control and Prevention has indicated that few of these developed methods are actually acceptable for diagnostic assessment [37, 44, 52]. Nonetheless, these amplification techniques have demonstrated higher sensitivities and specificities compared to other diagnostics and have emerged as the new standard for *M. pneumoniae* pneumonia detection in HIV [44].

### Coxiella burnetii

*Coxiella burnetii* (Q fever) is an obligate intracellular bacterium capable of causing acute and chronic illnesses in both immunocompromised and immunocompetent individuals alike [53]. However, reports of HIV-associated Q fever pneumonia are currently limited. Of those that have been reported, majority of them are from the pre-cART era [25, 30, 53, 54]. Nevertheless, information from the pre-cART era allows us to make inferences as to how *C. burnetii* will affect untreated HIV-infected patients, and those that have previously been treated with cART but have already progressed to AIDS.

In the 1990s, Q fever pneumonia rates were 0.3% in the general population and 9.7% in the untreated HIV-seropositive adult population. During this time, HIV-infected individuals were reported to be 13 times more likely to contract Q fever than healthy individuals [54].

Like the other atypical pneumonias, the clinical course of *C. burnetii* pneumonia is similar in both HIV-positive and negative individuals [53]. Symptoms can last up to 10 days and are often non-specific (e.g. fever, headache, non-productive cough, myalgia); however, in 90% of cases involving HIV, lung nodules have been shown to occur.

Diagnosing HIV-associated Q fever pneumonia can be quite challenging due to the many clinical forms of the disease (e.g. acute or chronic pulmonary infection) and to the decreasing immunity associated with HIV [54]. Diagnosis is based on serology and NAAT, however the potential of false negatives seen in serology increases with HIV disease advancement [25, 30, 54]. Moreover, in Q fever endemic areas, single serum samples may result in false positives, thus convalescent serum samples may be required. NAAT, and, more specifically, PCR—a more promising alternative with high specificity—are not widely available [55, 56]. Due to the lack of knowledge regarding when to test for HIV-associated Q fever pneumonia, *C. burnetii* diagnostics in HIV-infected patients are infrequently attempted and are likely to be underrepresented [30, 54, 57].

### Legionella pneumophila

The opportunistic intracellular pathogen *Legionella pneumophila* is a particular problem in immunosuppressed patients, and is estimated to be responsible for 20% of all adult HIV-associated pneumonias (compared to 15% in the general population), although surprisingly very few cases have actually been reported to date [15, 58–64]. Of the cases that have been recorded, many have shown that HIV-infected patients (particularly those with advanced immunosuppression) often present with a more severe clinical presentation compared to normal individuals [4, 58, 65].

In general, *L. pneumophila* pneumonia symptoms are non-specific with significantly higher rates of cough, dyspnea, bilateral pulmonary involvement, and hyponatremia in people with HIV [58, 62, 66, 67]. However, atypical manifestations involving the gastrointestinal tract or the central nervous system may also occur, making initial diagnosis quite challenging [62, 66, 67]. Recurrent lung cavitation has been shown to occur almost exclusively in immunosuppressed patients and often occurs shortly after initiation of therapy [58, 60, 68]. Complications due to respiratory failure and higher mortality rates have also been seen [67].

*L. pneumophila* infections may be underrepresented in the HIV population due to the fact that routine screening for *Legionella* is not usually performed and requires a special request from the clinician [4, 63]. Diagnosing HIV-associated *L. pneumophila* pneumonia has traditionally been reliant on culture and the urinary antigen test [62, 69]; however, culture requires specialized media, several days for growth, and still only has about 80% sensitivity [70]. For serology, depending on the severity of the patient’s immunosuppression, measurable *L. pneumophila* antigen may not be detectable initially, resulting in a false negative for the urinary antigen test [62, 66]. In a case by Franzin et al. [62], a negative urinary antigen result postponed *L. pneumophila* diagnosis in a cART-adhering, HIV-infected adult male until cultures were obtained (several days later). Thus, definitive diagnosis of HIV-associated *L. pneumophila* pneumonia has been reliant on two methods, both known to have their own respective limitations [62, 69]. Consequently, NAAT has become the new standard in diagnostics. Real-time PCR methods, targeting the *Legionella* mip gene, are considered to be more specific, sensitive and rapid compared to traditional diagnostics (with approximately a 15% increased yield over culture) and have been adapted for use in multiple laboratories; however, in developing nations, these automated techniques are not readily available [17, 71, 72]. Lastly, HIV patients are often co-infected with more than one pathogen which could mask infection with *L. pneumophila*. Consequently, *L. pneumophila* may play a much larger role in HIV-associated pneumonia than is currently anticipated.

### Non-pneumophila *Legionellas*

Pneumonia by other non-pneumophila *Legionella* species accounts for 10% of all legionellosis in the general population (with *Legionella micdadei* and *Legionella bozemanae* accounting for 90% of these cases) with limited information regarding these infections in HIV. However, of the information that has been gathered, it seems that cART-adhering HIV-infected individuals have higher rates of non-pneumophila pneumonia than healthy individuals [73–75].

In both treated and untreated HIV, *Legionella* non-pneumophila infections commonly manifest as fever, cough, dyspnea, diarrhea, pleuritic chest pain, and effusion, with documented instances of pulmonary cavities, nodules, and lung abscesses [73, 75–77]. Studies from the pre-cART era indicate that higher mortality rates are associated with infection in untreated HIV, although this may be due to the fact that these infections have only been reported in severely immunosuppressed patients and may not be due to the virulence of the microbes themselves [77–79].

Diagnosis of HIV-associated non-pneumophila *Legionella* pneumonia requires high clinical suspicion. Until a definitive diagnosis is reached, aggressive empirical therapy should be administered, especially in immunodeficient patients, in order to ensure a more positive outcome. Indeed*,* discontinuing empiric therapy in an immunocompromised adult HIV-infected individual despite a high suspicion of *Legionella* infection can lead to fatality [77].

Currently, culture is the best at diagnosing non-pneumophila pneumonia in HIV; however, sensitivities vary depending on the laboratory, with higher sensitivities having only been recorded in laboratories with a special interest in legionellosis [73, 75–77, 80, 81]. Urinary antigen, although useful for *L. pneumophila* serogroup 1 detection, is less sensitive for other serogroups and is practically useless for non-pneumophila species [77, 82]. NAAT methods, specifically PCR of lower respiratory tract specimens, have demonstrated high sensitivities (up to 100%) with *Legionella* species and may be a possible alternative for detecting non-pneumophila *Legionella* pneumonia in HIV-infected patients. However, although PCR assays can detect all of the various *Legionella* species with high specificity, they are currently not readily available for clinical use [17].

Little is known about non-pneumophila pneumonia and its prevalence in HIV, which may simply be due to the fact that *L. pneumophila* serogroup 1 is typically the only *Legionella* species that is often considered; the urinary antigen test targets *L. pneumophila* serogroup 1 and so do many serological assays [76]. The distribution of *Legionella* varies globally, therefore the usefulness of the urinary antigen test should be validated in each locale [83]. Moreover, HIV-associated legionellosis due to non-pneumophila is similar to *L. pneumophila*, which could prevent differentiation between these infections. To better determine the role of these pathogens in HIV infection, further development of more appropriate diagnostic techniques and increased clinical awareness are required.

### Tropheryma whipplei

*Tropheryma whipplei*, although not usually considered one of the atypical bacteria, has been found in respiratory samples of treated HIV-infected individuals at higher prevalence rates than the general population [84–87]. Currently, it is unclear whether *T. whipplei* is a pneumonia-causing pathogen or merely a commensal organism, since it has been found in both symptomatic and asymptomatic cases alike [84, 88–90]. Although some studies report *T. whipplei* as a pathogen (and even attribute certain clinical manifestations to this bacterium), caution is required until more evidence is acquired about the role of this microbe in HIV-associated pneumonia.

### Treatment of atypical bacterial pneumonia in HIV

Unlike typical bacterial pneumonia, atypical bacterial pneumonia does not respond to beta-lactams, aminoglycosides, and sulfa drugs; therefore, a 7–10 day course of macrolides (clarithromycin, erythromycin, or azithromycin), doxycycline and/or fluoroquinolones (levofloxacin or moxifloxacin) are required to treat these infections in HIV patients [29, 57, 62, 66, 91].

## Conclusions

Bacterial pneumonia is an immense problem among immunocompromised HIV-infected individuals, contributing to the high morbidity and eventual death of these patients. Although a large proportion of pneumonia is attributable to typical bacterial infections, clinicians must be aware of other relevant pathogens, such as *C. pneumoniae*, *M. pneumoniae*, *C. burnetti*, *Legionella* species, and, possibly, *T. whipplei*.

Diagnosing HIV-associated atypical pneumonia remains a challenging task and becomes even more so when the patient is severely immunocompromised. Due to the lack of data, the lack of consideration, and the current subpar diagnostic methods, atypical bacterial pneumonia is often left undiagnosed in HIV-infected individuals. Studies using more invasive methods (e.g. bronchoscopy and BAL) may provide a more accurate depiction of pneumonia. Further studies, and the development of more appropriate diagnostic methods, are required to clarify the role and prevalence rates of atypical bacterial pneumonia in HIV.

## Abbreviations

AIDS: Acquired immunodeficiency syndrome
BAL: Bronchoalveolar lavage
cART: Combination antiretroviral therapy
HIV: Human immunodeficiency virus
IFA: Immunofluorescence assay
MIF: Microimmunofluorescence
NAAT: Nucleic acid amplification techniques
PCR: Polymerase chain reaction
